# Risks of myeloid malignancies in patients with autoimmune conditions

**DOI:** 10.1038/sj.bjc.6604935

**Published:** 2009-03-03

**Authors:** L A Anderson, R M Pfeiffer, O Landgren, S Gadalla, S I Berndt, E A Engels

**Affiliations:** 1 Centre for Public Health, Queen's University Belfast, Northern Ireland BT12 6BJ, UK; 2 Division of Cancer Epidemiology and Genetics, National Cancer Institute, Rockville, MD 20892, USA; 3 Medical Oncology Branch, Center for Cancer Research, National Cancer Institute, Bethesda, MD, USA

**Keywords:** autoimmune conditions, myeloid leukaemia, myelodysplastic syndrome, chronic myeloproliferative diseases

## Abstract

Autoimmune conditions are associated with an elevated risk of lymphoproliferative malignancies, but few studies have investigated the risk of myeloid malignancies. From the US Surveillance Epidemiology and End Results (SEER)-Medicare database, 13 486 myeloid malignancy patients (aged 67+ years) and 160 086 population-based controls were selected. Logistic regression models adjusted for gender, age, race, calendar year and number of physician claims were used to estimate odds ratios (ORs) for myeloid malignancies in relation to autoimmune conditions. Multiple comparisons were controlled for using the Bonferroni correction (*P*<0.0005). Autoimmune conditions, overall, were associated with an increased risk of acute myeloid leukaemia (AML) (OR 1.29) and myelodysplastic syndrome (MDS, OR 1.50). Specifically, AML was associated with rheumatoid arthritis (OR 1.28), systemic lupus erythematosus (OR 1.92), polymyalgia rheumatica (OR 1.73), autoimmune haemolytic anaemia (OR 3.74), systemic vasculitis (OR 6.23), ulcerative colitis (OR 1.72) and pernicious anaemia (OR 1.57). Myelodysplastic syndrome was associated with rheumatoid arthritis (OR1.52) and pernicious anaemia (OR 2.38). Overall, autoimmune conditions were not associated with chronic myeloid leukaemia (OR 1.09) or chronic myeloproliferative disorders (OR 1.15). Medications used to treat autoimmune conditions, shared genetic predisposition and/or direct infiltration of bone marrow by autoimmune conditions, could explain these excess risks of myeloid malignancies.

The aetiology of myeloid malignancies, a heterogeneous group of disorders including acute myeloid leukaemia (AML), chronic myeloid leukaemia (CML), myelodysplastic syndrome (MDS) and chronic myeloproliferative disorders (MPDs), remains largely unknown. The main risk factors identified for AML are cigarette smoking (Kasim *et al*, 2005; Xu *et al*, 2007) and exposure to benzene or ionising radiation (Descatha *et al*, 2005). Chronic myeloid leukaemia arises from a translocation t(9;22)(q34;q11), known as the Philadelphia chromosome, and is thought to be associated with radiation exposure (Preston-Martin *et al*, 1989). Myelodysplastic syndrome is a heterogeneous clonal haematological disorder that can progress to AML. Cigarette smoking (Nisse *et al*, 2001), solvent exposure (Nisse *et al*, 2001) and agricultural exposures (Strom *et al*, 2005) have all been associated with an increased risk of MDS, and senescence of the haematopoietic system is thought to play a role in its development (Dalamaga *et al*, 2002). Less is known about the causes of MPDs, including polycythemia vera and essential thrombocythemia.

Immune perturbations, including autoimmune diseases, have been associated with an increased risk of haematological malignancies. Although reported more commonly for lymphoproliferative neoplasms (Zintzaras *et al*, 2005; Ekstrom Smedby *et al*, 2008), increased risks in persons with autoimmune conditions have also been noted for myeloid malignancies, including AML and CML (Zheng *et al*, 1993; Askling *et al*, 2005b). Autoimmune conditions, which occur commonly in patients with MDS (Voulgarelis *et al*, 2004), have also been reported to precede MDS (Dalamaga *et al*, 2002). Using data from the Surveillance Epidemiology and End Results (SEER)-Medicare Assessment of Hematopoietic Malignancy Risk Traits (SMAHRT) study, we investigated whether autoimmune conditions were associated with subsequent risks of AML, CML, MDS and MPDs.

## Materials and methods

The SMAHRT study is a population-based case–control study of haematopoietic malignancies using SEER-Medicare data (Warren *et al*, 2002; Anderson *et al*, 2008). The SEER program collects data on cancer diagnoses from multiple US sites since 1973 and currently covers ∼25% of the US population (Warren *et al*, 2002). Medicare provides federally funded health insurance for persons aged 65 years and older in the United States. All Medicare beneficiaries are entitled to Part A coverage, which includes hospital inpatient care. Approximately 96% of participants subscribe to Part B coverage, which covers both physician and outpatient services. The SEER-Medicare database has demographic and clinical information from SEER on cancer patients through December 2002, linked to their Medicare enrolment and claims data (Part A claims: 1986–2002; Part B claims: 1991–2002) (Warren *et al*, 2002). In addition, the Medicare data are available for a 5% random sample of all Medicare beneficiaries without cancer residing in SEER areas.

The SMAHRT study includes, as cases, those individuals with a SEER diagnosis of a first primary haematopoietic malignancy (*n*=93 780) between 1987 and 2002. We restricted this analysis to persons with a myeloid malignancy (*n*=13 486). Cases were classified into the following categories: AML (ICD-03 codes: 9840, 9861, 9866, 9867, 9870–9874, 9891, 9895–9897, 9910, 9920, 9930, 9931), CML (ICD-03: 9863, 9875, 9876), MDS (ICD-03: 9945, 9980, 9982, 9983, 9985, 9986, 9989) and MPD (ICD-03: 9741, 9742, 9750, 9754, 9755, 9756, 9757, 9950, 9960, 9961–9964, 9975). As MDS and MPD were not reportable to SEER registries until 2001 (Jaffe *et al*, 2001), only cases diagnosed in 2001 and 2002 were included (*n*=2471 and *n*=1017, respectively). Cases were required to be aged 67–99 years at diagnosis of malignancy and to have at least 12 months of Part A, Part B and non-HMO Medicare coverage before diagnosis to ensure adequate time for accrual of Part A (and where applicable Part B) Medicare claims to ascertain the occurrence of autoimmune conditions. Persons diagnosed with malignancy only at autopsy or by a death certificate and those with human immunodeficiency virus infection were excluded.

The SMAHRT study includes two controls per haematopoietic malignancy case, selected from the 5% random sample of Medicare beneficiaries who were alive, free of any malignancy, and had at least 12 months of prior Medicare coverage as of 1 July in the calendar year of selection. Controls were frequency matched to haematopoietic malignancy cases by the calendar year of diagnosis, age in five categories (67–69, 70–74, 75–79, 80–84 and 85–99 years) and gender. A person could be selected multiple times as a control for cases in different calendar years. All the SMAHRT study controls (*n*=160 086) were included in the AML and CML analyses. Only controls selected in 2001 and 2002 (*n*=42 886) were included in the MDS and MPD analyses.

We used hospital, physician and outpatient Medicare claims to assess whether individuals had any of the 27 specified autoimmune conditions (see Table 2). In assessing exposures, we required that the autoimmune condition was specified on at least one hospital claim, or at least two-physician or outpatient claims at least 30 days apart. Claims occurring during the 12-month period before case diagnosis–control selection were excluded to minimise the possibility that diagnoses of autoimmune conditions would be over-represented in cases because of ascertainment during the work-up for the malignancy.

### Statistical analysis

Unconditional logistic regression was used to calculate the odds ratios (ORs) and 95% confidence intervals (CIs), to assess the association of autoimmune conditions with myeloid malignancies separately for cases of each type compared with that in controls. We accommodated in the variance computation for the ORs the fact that each case subtype was compared with the same control population, that some controls later served as cases and the repeat selection of individuals as controls (Anderson *et al*, 2008). The AML and CML analyses were adjusted for age (using the categories of 67–69, 70–74, 75–79, 80–84, and 85–99 years), gender, year of diagnosis/selection (1987–1996, 1997–1999, 2000–2001, 2002), race (white, other/unknown) and, as a measure of overall healthcare utilisation, the number of physician claims more than 12 months before diagnosis/selection (quartiles: 0–20, 21–57, 58–127, ⩾128). The MDS and MPD analyses were adjusted for the same factors, except that the year of diagnosis/selection was included in models as two categories (2001 and 2002). As some myeloid malignancies, particularly MDS and MPD, can be chronic conditions, we conducted additional analyses excluding the 2 and 5 years before the diagnosis date of cases and the selection date of controls to ensure that the autoimmune conditions preceded MDS and MPD.

As we conducted 108 separate analyses to investigate the associations between 27 autoimmune conditions and 4 myeloid malignancies, we considered the impact of multiple testing on our findings. Although we indicate associations significant at a nominal significance level of *P*=0.05, we especially highlight in the tables and in the Discussion section those associations that were significant at a *P*-value of 0.0005, which incorporates a Bonferroni correction.

## Results

This study included 13 486 cases with a myeloid malignancy, which comprised 7824 individuals with AML, 2174 with CML, 2471 with MDS and 1017 with MPD. Compared with controls, cases were more likely to be male (except for MPD, in which cases were more likely to be female) and of white race. The median age at diagnosis/selection was similar for the myeloid malignancy cases and controls. For each myeloid neoplasm, cases had more prior physician, outpatient and hospital claims than controls (Table 1).

As shown in Table 2, having any autoimmune condition was associated with an increased risk of AML (OR 1.29). Specifically, AML was associated positively with several autoimmune conditions at the *P*<0.05 significance level, including rheumatoid arthritis (OR 1.28), systemic lupus erythematosus (OR 1.92), polymyalgia rheumatica (OR 1.73), autoimmune haemolytic anaemia (AIHA) (OR 3.74), systemic vasculitis (OR 6.23), giant cell arteritis (OR 1.61), ulcerative colitis (OR 1.72) and pernicious anaemia (OR 1.57). Chronic myeloid leukaemia was increased with polymyalgia rheumatica (OR 1.79), dermatomyositis/polymyositis (OR 3.97), AIHA (OR 5.23) and coeliac disease (OR 4.19) (Table 2).

Overall, MDS was associated with having an autoimmune condition (OR 1.50) and specifically with rheumatoid arthritis (OR 1.52), Sjögren's syndrome (OR 1.78), systemic lupus erythematosus (OR 1.82), polymyalgia rheumatica (OR 1.47), AIHA (OR 4.12), chronic rheumatic heart disease (OR 1.28), polyarteritis nodosa (OR 4.31), discoid lupus erythematosus (OR 2.06) and pernicious anaemia (OR 2.38). In comparison, MPD was only associated with AIHA (OR 11.9), localised scleroderma (OR 2.34) and Crohn's disease (OR 2.18).

As MDS and MPD are often chronic diseases that may go unrecognised for several years, we conducted a sensitivity analysis excluding claims within 2 or 5 years of diagnosis. Compared with controls, MDS cases more commonly had rheumatoid arthritis (OR 1.52), polymyalgia rheumatica (OR 1.53) and pernicious anaemia (OR 1.68) reported more than 5 years before the diagnosis/selection (Table 3). Similiarly, localised scleroderma remained associated significantly with MPD when the 2-year or 5-year period before diagnosis was excluded (ORs 2.73 and 3.27, respectively) (Table 3).

Associations that remained significant after using a Bonferroni correction (*P*<0.0005) are highlighted in Table 2. These included rheumatoid arthritis, systemic lupus erythematosus, polymyalgia rheumatica, AIHA, systemic vasculitis, ulcerative colitis and pernicious anaemia with AML; dermatomyositis/polymyositis with CML; rheumatoid arthritis and pernicious anaemia with MDS; and AIHA with MPD.

## Discussion

In this large study of 13 486 individuals with a myeloid malignancy, we investigated systematically the associations with a range of preceding autoimmune conditions. Overall, having an autoimmune condition was associated with an increased risk of AML and MDS, but not CML or MPD. Given the large number of comparisons, we focus here on associations that were significant at a *P*-value cutoff provided by the Bonferroni method (*P*<0.0005).

We observed significantly increased risks of AML and MDS associated with rheumatoid arthritis. Supporting this observation, Askling *et al* (2005b) found two-fold increased risks of AML and CML in a large Swedish cohort study of patients hospitalised for rheumatoid arthritis. Although MDS is a chronic condition, we saw an association with rheumatoid arthritis even after excluding the 5-year period before diagnosis/selection, arguing against reverse causality (i.e., that undiagnosed MDS caused rheumatoid arthritis). Several case reports have described AML and MDS occurring in patients with rheumatoid arthritis, mainly after treatment with azathioprine (Alexson and Brandt, 1977; Kwong *et al*, 1998) or methotrexate (Espinosa *et al*, 2002; Okamoto *et al*, 2004). However, non-steroidal anti-inflammatory drugs, used in the treatment of rheumatoid arthritis and other inflammatory conditions included in our study, appear to decrease the risk of myeloid leukaemia (Pogoda *et al*, 2005), suggesting that their use is unlikely to explain the excess risk of myeloid malignancies. Unfortunately, we lacked treatment information and were, therefore, unable to determine whether the observed increased risks of AML and MDS were related to therapy.

We also observed an increased risk of AML with systemic lupus erythematosus, which might be attributed partly to the use of immune-modulating treatments, such as azathioprine (Alexson and Brandt, 1977; Kwong *et al*, 1998). Another systemic autoimmune condition associated with an increased risk of AML and CML (and to a lesser extent MDS) was polymyalgia rheumatica. Some studies have reported polymyalgia rheumatica to occur subsequent to diagnosis of MDS (Mok *et al*, 1996; Espinosa *et al*, 2002), and polymyalgia rheumatica has been reported to precede AML (Anton, 2007). Giant cell arteritis, a condition closely related to polymyalgia rheumatica, was more weakly associated with AML in our study. Among the other autoimmune conditions affecting the cardiovascular system, only systemic vasculitis remained associated with AML after adjustment for multiple comparisons. To our knowledge, AML has not been reported in patients with systemic vasculitis, though large-vessel arteritis has been reported in patients with MDS (Steurer *et al*, 2004).

In our study, AIHA significantly increased the risk for all of the myeloid malignancies. Autoimmune haemolytic anaemia is considered to be a complication of several lymphoproliferative disorders (Ekstrom Smedby *et al*, 2008). Although case reports describe AIHA with MDS (Giagounidis *et al*, 2005), AML (Deutsch *et al*, 2003) and CML (Arbaje and Beltran, 1990), we found AIHA to occur antecedent to these diagnoses. Consistent with our findings, AIHA was found to be associated with an eight-fold increased risk of AML in a large cohort study in Sweden (Soderberg *et al*, 2006). As MDS and MPD are indolent and may be present years before diagnosis, it is possible that AIHA arose as a result of these conditions. This explanation is less likely for AML and CML, and it is possible that AIHA acts late in a causal pathway to promote the development of these malignancies.

Ulcerative colitis was associated with an increased risk of AML, in keeping with a Swedish population-based cohort study which found an 80% increase in ulcerative colitis patients (Askling *et al*, 2005a). However, other cohort studies found no such relation between ulcerative colitis and risk of AML (Bernstein *et al*, 2001; Winther *et al*, 2004; Hemminki *et al*, 2008). Pernicious anaemia has been reported antecedent to AML (Hsing *et al*, 1993), consistent with the association we observed. Dermatomyositis/polymyositis was associated strongly with CML. Although these conditions manifest commonly in cancer patients (Stockton *et al*, 2001), the association with CML remained elevated even when the 5-year period before diagnosis was excluded, indicating that dermatomyositis/polymyositis may also precede CML.

There are several possible explanations for these associations with myeloid malignancies. First, as mentioned earlier, certain treatments for autoimmune conditions, such as azathioprine, could increase the risk of developing MDS or AML. However, the associations with AML were not specific to autoimmune conditions treated with these medications. Second, some autoimmune conditions could share common genetic predispositions with myeloid malignancies. For example, carriers of the human leucocyte antigen-B27 are predisposed to some autoimmune conditions and AML (Au *et al*, 2001). Polymorphisms of interleukin-1 have been associated with several autoimmune conditions, and polymorphisms in the interleukin 1 receptor antagonist are associated with AML (Demeter *et al*, 1996). Finally, an intriguing possibility is that autoimmune conditions could infiltrate the bone marrow and cause damage to the myeloid precursor cells that differentiate into blood cells.

The SMAHRT study has several strengths including the large number of individuals with myeloid malignancies, the population-based sampling of cases and the random selection of controls from the population. The SEER database covers ∼25% of the US population (Warren *et al*, 2002) making our study representative of the elderly US population. In addition, the availability of outpatient, inpatient and physician claims allowed us to investigate the associations between a range of autoimmune conditions and myeloid malignancies. Our study also has some limitations. First, the small number of cases and controls with some uncommon autoimmune conditions limit the precision of our estimates, so cautious interpretation is indicated. Second, as claim files were utilised in place of a definite diagnosis, autoimmune conditions which required few physician visits could have been underestimated. Third, despite our exclusion of a 1-year period before cancer diagnosis, some autoimmune conditions could have been the result of the myeloid malignancy. This seems unlikely for AML and CML, but could explain some associations with the more indolent conditions, MDS and MPD. Fourth, as MDS and MPD are a heterogeneous group of diseases, some associations could have been masked by combining these conditions into one category. Unfortunately, we did not have sufficient sample sizes to investigate risk by more specific subtypes. Fifth, cases and controls differed according to some factors, such as race and frequency of Medicare claims, which could have led to differences in the prevalence of autoimmune conditions or our ability to detect their presence. However, we adjusted for these differences in our statistical models. Finally, as some of the numerous associations investigated may have occurred by chance, we therefore used a Bonferroni correction to highlight those associations least likely to be due to chance.

In summary, certain autoimmune conditions were associated with increased risks of MDS, MPD, CML, and in particular AML, possibly due to a common genetic predisposition, the effects of medications used to treat autoimmune conditions, or direct damage of the bone marrow by autoimmune conditions.

## Figures and Tables

**Table 1:** Characteristics of cases with myeloid malignancies and controls in the SMAHRT study

	**Selection years 1987–2002**			**Selection years 2001–2002**		
**Characteristics**	**Controls (*n*=160 086)**	**Acute myeloid leukaemia (*n*=7824)**	**Chronic myeloid leukaemia (*n*=2174)**	**Controls (*n*=42 886)**	**Myelodysplastic syndromes^a^ (*n*=2471)**	**Chronic myeloproliferative disorder^b^ (*n*=1017)**
*Gender*						
Male	78 620 (49.1%)	4156 (53.1%)	1122 (51.6%)	21 460 (50.0%)	1339 (54.2%)	457 (44.9%)
Female	81 466 (50.9%)	3668 (46.9%)	1052 (48.4%)	21 426 (50.0%)	1132 (45.8%)	560 (55.1%)
						
*Age, years*						
67–69	19 135 (12.0%)	893 (11.4%)	278 (12.8%)	4085 (9.5%)	156 (6.3%)	95 (9.3%)
70–74	40 611 (25.4%)	1957 (25.0%)	501 (23.1%)	9788 (22.8%)	472 (19.1%)	232 (22.8%)
75–79	41 724 (26.1%)	2050 (26.2%)	546 (25.1%)	11 330 (26.4%)	651 (26.4%)	285 (28.0%)
80–84	32 091 (20.1%)	1627 (20.8%)	433 (19.9%)	9742 (22.7%)	654 (26.5%)	239 (23.5%)
85–99	26 525 (16.6%)	1297 (16.6%)	416 (19.1%)	7941 (18.5%)	538 (21.8%)	166 (16.3%)
Median (range)	77 (67–99)	77 (67–99)	77 (67–99)	78 (67–99)	78 (67–99)	79 (67–99)
						
*Selection year*						
1987–1996	71 396 (44.6%)	3355 (42.9%)	1173 (54.0%)	—	—	—
1997–1999	26 946 (16.8%)	1411 (18.0%)	324 (14.9%)	—	—	—
2000	40 750 (25.5%)	2105 (26.9%)	484 (22.3%)	—	—	—
2001–2002	20 994 (13.1%)	953 (12.2%)	193 (8.9%)	42 886 (100%)	2471 (100%)	1017 (100%)
						
*Race/ethnicity*						
White	135 280 (84.5%)	6912 (88.3%)	1900 (87.4%)	35 959 (83.9%)	2173 (87.9%)	875 (86.0%)
Black	10 897 (6.8%)	386 (4.9%)	148 (6.8%)	2973 (6.9%)	156 (6.3%)	85 (8.4%)
Asian	5629 (3.5%)	160 (2.0%)	39 (1.8%)	1689 (3.9%)	62 (2.5%)	21 (2.1%)
Hispanic	3408 (2.1%)	88 (1.1%)	31 (1.4%)	1157 (2.7%)	40 (1.6%)	19 (1.9%)
Native American Indian	448 (0.3%)	7 (0.1%)	4 (0.2%)	111 (0.3%)	5 (0.2%)	2 (0.2%)
Other/unknown	4424 (2.8%)	271 (3.5%)	52 (2.4%)	997 (2.3%)	35 (1.4%)	15 (1.5%)
						
*Duration of Medicare coverage* c						
13–57 months	62 264 (38.9%)	2186 (36.0%)	919 (42.3%)	10 208 (23.8%)	453 (18.3%)	228 (22.4%)
58–93 months	36 842 (23.0%)	1794 (22.9%)	504 (23.2%)	7312 (17.1%)	360 (14.6%)	163 (16.0%)
94–136 months	30 696 (19.2%)	1704 (21.8%)	430 (19.8%)	8571 (20.0%)	450 (18.2%)	204 (20.1%)
⩾137 months	30 284 (18.9%)	1510 (19.3%)	321 (14.8%)	16 795 (39.2%)	1208 (48.9%)	422 (41.5%)
						
*Number of physician claims* d						
0–20	68 324 (42.7%)	2931 (37.5%)	952 (43.8%)	8285 (19.3%)	280 (11.3%)	143 (14.1%)
21–57	30 532 (19.1%)	1329 (17.0%)	382 (17.6%)	7949 (18.5%)	307 (12.4%)	199 (19.6%)
58–127	30 763 (19.2%)	1548 (19.8%)	381 (17.5%)	10 843 (25.3%)	568 (23.0%)	272 (26.8%)
⩾128	30 467 (19.0%)	2016 (25.8%)	459 (21.1%)	15 809 (36.9%)	1316 (53.3%)	403 (39.6%)
						
*Number of outpatient claims* d						
0	62 453 (39.0%)	2736 (35.0%)	880 (40.5%)	7323 (17.1%)	253 (10.2%)	109 (10.7%)
1–3	32 154 (20.1%)	1405 (18.0%)	403 (18.5%)	8404 (19.6%)	332 (13.4%)	164 (16.1%)
4–7	21 293 (13.3%)	1042 (13.3%)	278 (12.8%)	7005 (16.3%)	339 (13.7%)	163 (16.0%)
8–15	20 722 (12.9%)	1106 (14.1%)	256 (11.8%)	8242 (19.2%)	498 (20.2%)	190 (18.7%)
⩾16	23 464 (14.7%)	1535 (19.6%)	357 (16.4%)	11 912 (27.8%)	1049 (42.5%)	391 (38.5%)
						
*Number of hospital claims* d						
0	87 059 (54.4%)	3658 (46.8%)	1001 (46.0%)	20 058 (46.8%)	803 (32.5%)	443 (43.6%)
1	28 505 (17.8%)	1533 (19.6%)	419 (19.3%)	7623 (17.8%)	449 (18.2%)	199 (19.6%)
2–3	25 255 (15.8%)	1447 (18.5%)	412 (19.0%)	7779 (18.1%)	561 (22.7%)	169 (16.6%)
⩾4	19 267 (12.0%)	1186 (15.2%)	342 (15.7%)	7426 (17.3%)	658 (26.6%)	206 (26.6%)

SMAHRT=Surveillance Epidemiology and End Results (SEER)-Medicare Assessment of Hematopoietic Malignancy Risk Traits.

a This category includes patients with refractory anaemia, refractory cytopenia with multilineage dysplasia, myelodysplastic syndrome with 5q deletion and myelodysplastic syndrome, not otherwise specified, diagnosed during 2001–2002.

b This category includes patients with chronic neutrophilic leukaemia, chronic esinophilic leukaemia, chronic myeloproliferative disease not otherwise specified, chronic idiopathic myelofibrosis, essential thrombocythemia, polycythemia vera, mastocytosis and neoplasms of histiocytes and accessory lymphoid cells diagnosed during 2001–2002.

c Duration of coverage refers to simultaneous coverage by Part A and Part B while the individual was not enroled in a health maintenance organisation.

d The number of claims excludes the 12 months before haematopoietic malignancy diagnosis (cases) or selection (controls).

**Table 2:** Associations between autoimmune conditions and risk of myeloid malignancies

	**Selection years 1987–2002**					**Selection years 2001–2002**				
	**Controls (*n*=160 086)**	**Acute myeloid leukaemia (*n*=7824)**		**Chronic myeloid leukaemia (*n*=2174)**		**Controls (*n*=42 886)**	**Myelodysplastic syndromes (*n*=2471)**		**Chronic myeloproliferative disorder (*n*=1017)**	
**Autoimmune conditions**	**No.**	**No.**	**OR (95% CI)^a^**	**No.**	**OR (95% CI)^a^**	**No.**	**No.**	**OR (95% CI)^b^**	**No.**	**OR (95% CI)^b^**
Any autoimmune condition	14 056	973	1.29 (1.20–1.39)	208	1.09 (0.94–1.27)	5968	574	1.50 (1.35–1.66)	171	1.15 (0.97–1.37)
*Systemic/connective tissue*										
Rheumatoid arthritis	3425	237	1.28 (1.11–1.47) c	56	1.23 (0.94–1.62)	1480	150	1.52 (1.27–1.81) c	39	1.01 (0.73–1.41)
Sjögren's syndrome	261	16	1.10 (0.66–1.82)	<5	1.14 (0.42–3.09)	120	15	1.78 (1.03–3.07)	<5	0.90 (0.29–2.85)
Systemic lupus erythematosus	298	31	1.92 (1.31–2.80) c	5	1.28 (0.52–3.12)	117	14	1.82 (1.04–3.16)	<5	0.31 (0.04–2.23)
Sarcoidosis	101	10	1.84 (0.95–3.56)	<5	0.76 (0.11–5.46)	42	<5	1.11 (0.34–3.61)	0	—
Systemic sclerosis	83	<5	0.90 (0.33–2.47)	<5	0.94 (0.13–6.80)	38	5	2.05 (0.80–5.25)	<5	0.93 (0.13–6.85)
Polymyalgia rheumatica	1288	125	1.73 (1.43–2.09) c	32	1.79 (1.25–2.57)	518	55	1.47 (1.11–1.96)	15	1.11 (0.66–1.86)
Ankylosing spondylitis	133	11	1.43 (0.76–2.68)	<5	0.53 (0.07–3.84)	59	5	1.18 (0.47–2.93)	<5	2.04 (0.64–6.55)
Dermatomyositis/polymyositis	135	7	0.91 (0.42–1.96)	7	3.97 (1.82–8.61) c	60	<5	0.46 (0.11–1.90)	<5	0.61 (0.08–4.43)
										
*Blood*										
Autoimmune haemolytic anaemia	52	11	3.74 (1.94–7.22) c	<5	5.23 (1.82–15.0)	20	6	4.12 (1.66–10.2)	6	11.9 (4.72–30.2) c
										
*Cardiovascular*										
Systemic vasculitis	27	10	6.23 (2.81–13.8) c	0	—	8	<5	3.49 (0.71–17.0)	0	—
Chronic rheumatic heart disease	4099	239	1.01 (0.88–1.15)	57	0.99 (0.76–1.30)	1839	173	1.28 (1.08–1.51)	46	0.98 (0.73–1.33)
Giant cell arteritis	427	37	1.61 (1.14–2.27)	<5	0.36 (0.09–1.44)	171	16	1.28 (0.76–2.16)	<5	0.44 (0.11–1.77)
Polyarteritis nodosa	35	<5	1.97 (0.68–5.72)	0	—	16	5	4.31 (1.51–12.3)	<5	2.51 (0.33–19.1)
										
*Endocrine*										
Addison's disease	196	12	1.05 (0.59–1.89)	<5	1.49 (0.55–4.04)	92	6	0.89 (0.39–2.03)	<5	0.84 (0.21–3.43)
Graves’ disease	360	21	1.04 (0.66–1.62)	8	1.65 (0.81–3.35)	150	5	0.49 (0.20–1.20)	5	1.26 (0.52–3.06)
Hashimoto's thyroiditis	290	17	1.06 (0.65–1.74)	<5	1.07 (0.40–2.88)	149	10	0.97 (0.51–1.86)	8	1.98 (0.96–4.08)
										
*Skin*										
Psoriasis	1543	95	1.07 (0.87–1.33)	19	0.91 (0.58–1.44)	689	57	1.16 (0.88–1.53)	23	1.32 (0.87–2.01)
Alopecia areata	99	6	1.16 (0.50–2.66)	<5	0.81 (0.11–5.76)	50	<5	1.23 (0.44–3.42)	<5	0.73 (0.10–5.29)
Pemphigus	26	<5	0.69 (0.09–5.13)	0	—	11	0	—	0	—
Localised scleroderma	178	11	1.11 (0.60–2.06)	<5	0.43 (0.06–3.07)	94	5	0.76 (0.31–1.89)	6	2.34 (1.02–5.37)
Discoid lupus erythematosus	149	5	0.61 (0.25–1.50)	<5	0.51 (0.07–3.65)	63	9	2.06 (1.02–4.17)	0	—
										
*Gastrointestinal*										
Coeliac disease	54	<5	0.63 (0.15–2.60)	<5	4.19 (1.30–13.5)	26	<5	0.51 (0.07–3.77)	0	—
Crohn's disease	316	26	1.43 (0.95–2.15)	<5	0.67 (0.21–2.09)	120	14	1.60 (0.91–2.78)	7	2.18 (1.01–4.71)
Ulcerative colitis	504	50	1.72 (1.28–2.31) c	5	0.72 (0.30–1.74)	229	22	1.33 (0.86–2.07)	7	1.18 (0.55–2.51)
Pernicious anaemia	2008	177	1.57 (1.34–1.84) c	21	0.74 (0.48–1.14)	886	148	2.38 (1.98–2.86) c	25	1.11 (0.74–1.67)
										
*Nervous system*										
Multiple sclerosis	185	7	0.68 (0.32–1.45)	<5	0.77 (0.19–3.12)	55	<5	0.55 (0.13–2.22)	0	—
Myasthenia gravis	115	8	1.20 (0.58–2.49)	0	—	54	<5	0.25 (0.04–1.86)	<5	2.26 (0.71–7.24)

CI=confidence interval; OR=odds ratio; SEER=Surveillance Epidemiology and End Results.

Observations, in which the number of exposed patients or controls is between one and four, are listed as ‘<5’ to reserve subjects’ anonymity, in accordance with the SEER-Medicare data use agreement. Associations significant at the *P*<0.05 level are underlined.

For consistency across tables, all ORs are shown to two decimal places (or three significant figures if the OR ≥10.0). Nonetheless, we note that many estimates are based on few exposed cases.

a ORs and 95% CIs are adjusted for age (67–69, 70–74, 75–79, 80–84 and 85–99 years), gender, selection year (1987–1996, 1997–1999, 2000–2001, 2002), race (white, non-white) and number of physician claims (0–20, 21–57, 58–127, ⩾128).

b ORs and 95% CIs are adjusted for age (67–69, 70–74, 75–79, 80–84 and 85–99 years), gender, selection year (2001, 2002), race (white, non-white) and number of physician claims (0–20, 21–57, 58–127, ⩾128).

c Association is significant at *P*<0.0005 (Bonferroni correction for 108 comparisons).

**Table 3:** Associations between selected autoimmune conditions and myelodysplastic syndrome and chronic myeloid disorder with exclusions of time intervals before diagnosis/selection

	**Time interval before diagnosis/control selection evaluated for autoimmune conditions**		
	**>1 year**	**>2 years**	**>5 years**
**Myeloid subtype and autoimmune condition**	**OR (95% CI)^a^**	**OR (95% CI)^a^**	**OR (95% CI)^a^**
*Myelodysplastic syndrome*			
Rheumatoid arthritis	1.52 (1.27–1.81)	1.53 (1.27–1.84)	1.52 (1.19–1.92)
Sjögren's syndrome	1.78 (1.03–3.07)	1.77 (0.98–3.16)	1.94 (0.91–4.13)
Systemic lupus erythematosus	1.82 (1.04–3.16)	1.87 (1.05–3.32)	1.96 (0.94–4.10)
Polymyalgia rheumatica	1.47 (1.11–1.96)	1.57 (1.17–2.10)	1.53 (1.05–2.22)
Autoimmune haemolytic anaemia	4.12 (1.66–10.2)	4.14 (1.64–11.9)	1.32 (0.16–10.6)
Chronic rheumatic heart disease	1.28 (1.08–1.51)	1.26 (1.05–1.51)	1.16 (0.90–1.49)
Polyarteritis nodosa	4.31 (1.51–12.3)	3.44 (0.90–13.1)	2.78 (0.55–14.1)
Discoid lupus erythematosus	2.06 (1.02–4.17)	1.51 (0.65–3.51)	1.58 (0.56–4.46)
Pernicious anaemia	2.38 (1.98–2.86)	2.20 (1.79–2.70)	1.68 (1.24–2.26)
			
*Chronic myeloproliferative disorder*			
Autoimmune haemolytic anaemia	11.9 (4.72–30.2)	10.1 (3.27–31.1)	4.02 (0.50–32.5)
Localised scleroderma	2.34 (1.02–5.37)	2.73 (1.18–6.32)	3.47 (1.24–9.77)
Crohn's disease	2.18 (1.01–4.71)	2.09 (0.91–4.80)	2.00 (0.72–5.50)

CI=confidence interval; OR=odds ratio.

For consistency across tables, all ORs are shown to two decimal places (or three significant figures if the OR ≥10.0). Nonetheless, we note that many estimates are based on few exposed cases.

Associations significant at the *P*<0.05 level are underlined.

a ORs and 95% CIs are adjusted for age (67–69, 70–74, 75–79, 80–84 and 85–99 years), gender, race (white, non-white), number of physician claims (0–20, 21–57, 58–127, ⩾128) and selection year (2001, 2002).
